# Disability, health and human development – Book review

**DOI:** 10.4102/ajod.v8i0.538

**Published:** 2019-08-26

**Authors:** Zuzana Matousova-Done

**Affiliations:** 1 Private, Cape Town, South Africa

As a nurse trained in intellectual disability in the United Kingdom – and currently working in South Africa – I found Sophie Mitra’s new book *Disability, Health and Human Development* informative, well researched and thought-provoking.

It proposes a new conceptual framework, the Human Development Model, for persons living with disabilities in low- and middle-income countries (LMICs) using the Washington Group Short Set of Questions on Disability – collected as part of the Living Standard Measure Study. This set of questions assesses disability in six domains: seeing, hearing, walking, concentrating or remembering, self-care and communicating. Data sets from Ethiopia, Malawi, Tanzania and Uganda are analysed and interpreted to offer a fresh outlook on the definition of disability, health deprivation and the causes and consequences for well-being in LMICs.

Importantly, Mitra disabuses the reader of the misconception that disability disproportionately affects high-income countries (HICs) because of longer life expectancies. She explains that *worldwide* populations are living longer and growing older; in HICs, the advances in healthcare have often been coupled with the promotion of inclusivity, provision of resources, education, adapted workplace environments and social security to give people with various degrees of disability better opportunities to achieve their full potential and to reach their goals, dreams and aspirations. By contrast, Mitra argues, many LMICs have developed policies and legislation on disabilities without fully considering the Conventions on the Rights of Persons with Disability (United Nations [UN] 2008), and these policies may not provide an adequate solution to better the quality of life of persons with disability, despite the improvements in certain health parameters.

Mitra highlights that this group of people in LMICs often remain highly marginalised within their communities and may have no access to social security programmes or workplaces or education that is adapted to meet their needs, thus trapping them in a cycle of poverty. Her data also suggest that those with disabilities who are female, over 65 years age and already living in low-income households are especially unlikely to break out of this cycle.

The Human Development Model that Mitra introduces is based on Amartya Sen’s capability approach, which addresses different challenges in welfare economics related to disability, including standard of living, poverty and well-being (Mitra 2017:11). The capability approach focuses on the ability of a person to live life and have reason to value (Wells 2012) through practical opportunities (called capabilities) and achievements (called functioning). Arising from this paradigm, Mitra (2017) offers a new definition of disability as:

a deprivation in terms of functionings and/or capabilities among persons with health deprivations…Health deprivation and disability result from the interaction of personal factors (e.g. sex and age), structural factors (e.g. policies, social attitudes and physical environment) and resources (e.g. asset, information). (p. 154)

Mitra then argues convincingly for the importance of a person-centred approach in LMICs and describes clearly the need for holistic care, inclusivity, accessibility, adapting the physical environment and the crucial role of policymakers in poor communities to relieve the ‘deprivation’ that she defines.

The book is clearly and logically set out as follows:

Chapter 1 introduces the research; it sets the tone for the book and hints at the research questions to which Mitra offers preliminary answers based on research results, implications and ideas for further research as offered in Chapter 7. Mitra includes readers that may skip the quantitative method section and accounts to wide audience of readers throughout the chapters. Chapter 2 offers the conceptual framework, introduces the Human Developmental Model and compares it to other disability models that have been applied in the disability context; Mitra uses this framework to formulate a particular theoretical link between poverty and disability. The empirical context, data collection, analysis, methods, literature review and results, as well as reasons for choosing the four countries investigated, are discussed in Chapters 3–6. For a wide disability audience, Mitra manages to garner and reinforce awareness regarding the prevalence and socio-economic status as mediated by inequality.

Chapter 7 summarises the main results in a more accessible discussion and considers the implications for policies, programmes and data.

Each chapter is formatted with an abstract and conclusion, which enables the reader to understand the main concepts and how they interlink across chapters. Each chapter also links explicitly to the Human Developmental Model such that the reader gains an integrated understanding of the model and its application. Furthermore, Mitra critically analyses each point and offers possible solutions to identified limitations.

What this book does particularly well is to explain how Mitra arrived at her revised definition of disability, with the use of short examples and scenarios to clarify the more conceptual aspects of the research.

One minor criticism: for a work that is conceptually and linguistically dense and which has a target audience that will contain many second- and third-language English speakers such as myself, it would have been more user-friendly if all the terms introduced in Chapter 2 were also compiled and summarised into a separate glossary for quicker reference when reading the rest of the book.

Overall, however, Mitra displays an impressive first-hand understanding of the issues around disability in LMICs and convincingly demonstrates both the hardships endured by disabled people in these communities and the need for a holistic person centred approach to their care.
